# Classification of Twitter Users Who Tweet About E-Cigarettes

**DOI:** 10.2196/publichealth.8060

**Published:** 2017-09-26

**Authors:** Annice Kim, Thomas Miano, Robert Chew, Matthew Eggers, James Nonnemaker

**Affiliations:** ^1^ Center for Health Policy Science and Tobacco Research RTI International Berkeley, CA United States; ^2^ Center for Data Science RTI International Research Triangle Park, NC United States; ^3^ Center for Health Policy Science and Tobacco Research RTI International Research Triangle Park, NC United States

**Keywords:** electronic cigarettes, social media, machine learning

## Abstract

**Background:**

Despite concerns about their health risks, e‑cigarettes have gained popularity in recent years. Concurrent with the recent increase in e‑cigarette use, social media sites such as Twitter have become a common platform for sharing information about e-cigarettes and to promote marketing of e‑cigarettes. Monitoring the trends in e‑cigarette–related social media activity requires timely assessment of the content of posts and the types of users generating the content. However, little is known about the diversity of the types of users responsible for generating e‑cigarette–related content on Twitter.

**Objective:**

The aim of this study was to demonstrate a novel methodology for automatically classifying Twitter users who tweet about e‑cigarette–related topics into distinct categories.

**Methods:**

We collected approximately 11.5 million e‑cigarette–related tweets posted between November 2014 and October 2016 and obtained a random sample of Twitter users who tweeted about e‑cigarettes. Trained human coders examined the handles’ profiles and manually categorized each as one of the following user types: individual (n=2168), vaper enthusiast (n=334), informed agency (n=622), marketer (n=752), and spammer (n=1021). Next, the Twitter metadata as well as a sample of tweets for each labeled user were gathered, and features that reflect users’ metadata and tweeting behavior were analyzed. Finally, multiple machine learning algorithms were tested to identify a model with the best performance in classifying user types.

**Results:**

Using a classification model that included metadata and features associated with tweeting behavior, we were able to predict with relatively high accuracy five different types of Twitter users that tweet about e‑cigarettes (average *F*_1_ score=83.3%). Accuracy varied by user type, with *F*_1_ scores of individuals, informed agencies, marketers, spammers, and vaper enthusiasts being 91.1%, 84.4%, 81.2%, 79.5%, and 47.1%, respectively. Vaper enthusiasts were the most challenging user type to predict accurately and were commonly misclassified as marketers. The inclusion of additional tweet-derived features that capture tweeting behavior was found to significantly improve the model performance—an overall *F*_1_ score gain of 10.6%—beyond metadata features alone.

**Conclusions:**

This study provides a method for classifying five different types of users who tweet about e‑cigarettes. Our model achieved high levels of classification performance for most groups, and examining the tweeting behavior was critical in improving the model performance. Results can help identify groups engaged in conversations about e‑cigarettes online to help inform public health surveillance, education, and regulatory efforts.

## Introduction

E‑cigarettes have gained popularity among adults and youth in recent years. Following sustained increases in the use of e-cigarettes by adults from 2010 to 2013 [1], the prevalence of adult e‑cigarette use plateaued at 3.7% in 2014 and was reported to be much higher among current cigarette smokers (15.9%) [2]. Despite the slight decline in the use of e‑cigarettes by youth from 2014 to 2015, e‑cigarettes remain the most commonly used tobacco product among the middle and high school students in the United States, with 16.0% reporting current use in 2015 [3,4]. Although the long-term health effects of e‑cigarette use are largely unknown, e‑cigarettes commonly contain nicotine, which has negative effects on the adolescent brain [5], along with a range of other chemicals that are harmful to human health [6-10]. In addition, youth who initiate nicotine use with e‑cigarettes may transition to combustible tobacco use [11-14], which has been identified as the leading preventable cause of death in the United States [15].

Concurrent with the rapid rise in e‑cigarette use, advertising and sharing of information about e‑cigarettes have proliferated in recent years. Although advertisements for tobacco products have been banned on television since 1971 in the United States, e‑cigarette advertising via television, magazines, outdoor, radio, and Web-based channels has increased dramatically between 2010 and 2013. Approximately 24 million adolescents were exposed to e‑cigarette advertising in 2014 [16]. In addition to traditional advertising platforms, e‑cigarette–related information and promotional material are widely available through e‑cigarette user forums, Web-based marketing, branded websites, and user-generated content on social media sites such as Twitter and YouTube [17,18].

Social media has become a particularly important platform for sharing information about e‑cigarettes. The majority of youth (81%) and adults (74%) in the United States use some form of social media [19-21], and the microblog, Twitter, has more than 316 million active users creating more than 500 million brief posts (called *tweets*) daily [22]. Twitter’s pervasiveness makes it a convenient tool for e‑cigarette manufacturers, enthusiasts, and advocates to promote e‑cigarettes actively to a wide audience. Some studies of the content of e‑cigarette–related tweets suggest that the overwhelming majority is commercial or promotional in nature [23-25], and many of these tweets offer discounts or free samples [24]. However, recent research suggests that many tweets reflect discussion of policies, personal experiences, and risks and benefits associated with e‑cigarette use among individuals and e‑cigarette proponents [26]. Another study found that although the majority of Twitter users engaged in social media conversations about e‑cigarettes are not affiliated with the e‑cigarette industry, e‑cigarette proponents (ie, e‑cigarette marketing or manufacturing representatives, advocates, and enthusiasts) tweet more frequently and are more likely to highlight favorable aspects of e‑cigarette use [27].

Monitoring trends in e‑cigarette–related social media activity requires timely assessment of the content of posts and the types of users generating the content to inform regulatory and surveillance efforts. In 2016, the Food and Drug Administration (FDA) finalized a rule extending the agency’s authority to regulate e‑cigarettes, which includes federal provisions requiring companies that sell e‑cigarettes to include warning statements about nicotine on advertising and promotional materials, including content on digital/social media. To ensure that e‑cigarette companies are complying with these advertising and labeling restrictions, FDA will need to identify and monitor websites and social media accounts maintained by these companies. Furthermore, as public health researchers continue to use social media data to track and understand emerging issues concerning e‑cigarettes, they will need to be able to distinguish between the content from individuals who may be the target of Web-based e‑cigarette advertising (eg, young adults) and the content from e‑cigarette companies, marketers, or spammers who may be posting content for commercial purposes. Such information could also be useful in the development and targeting of social media campaigns to prevent e‑cigarette use.

The proliferation and variety of Web-based information sharing about e‑cigarettes presents challenges in differentiating content from different types of social media users. Previous studies have used a range of techniques to identify Twitter accounts that are purely automated (*robots*), human-assisted automated (*cyborgs*), or organic (ie, individuals) [28] and to distinguish between promotional and nonpromotional tweets [25,29]. Less is known about identifying the diversity of user types responsible for generating e‑cigarette–related content on Twitter, including vape proponents, promotional marketers, automated spammers, public health agencies, news organizations, and individuals. In a recent study of tweets about e‑cigarettes, Lazard and colleagues [26] analyzed clusters of e‑cigarette topics (eg, marketing-focused personal experience) to categorize tweets as being generated by marketers, individual users, or e‑cigarette proponents. However, this assessment was based on a review of the topics being discussed (eg, personal experience about e‑cigarette use must be posted by individual users) and was not informed by analysis of user handles that were tweeting the content. Thus, Lazard and colleagues’ attribution of message source may be limited. For example, Lazard and colleagues reported that tweets about e‑cigarette policy bans (a common topic cluster identified in the study) were posted by e‑cigarette proponents opposing the ban, but these tweets could have been posted by policy makers announcing or promoting the ban. Examining the topic of tweets may not be sufficient for attributing the source of the message. A more detailed assessment of Twitter users’ profile and tweet metadata, in addition to the content of their tweets, could provide better insights into the types of users posting the content.

This study demonstrates a novel methodology for automatically classifying Twitter users who tweet about e‑cigarette–related topics into five categories of users—individuals, vaper enthusiasts, informed agencies, marketers, and spammers. We used a supervised machine learning approach to predict different types of Twitter users based on their metadata and tweeting behavior. We tested different models, evaluated model performance, and discussed features that are most predictive of each user type. This study expands on previous research studying the content and the types of users who tweet about e‑cigarettes [23-25,27] by providing a greater level of granularity in the classification of users. Findings from this study provide insight into the composition and the characteristics of social media users posting about e‑cigarettes, which can help inform future regulatory action.

## Methods

**Figure 1:**
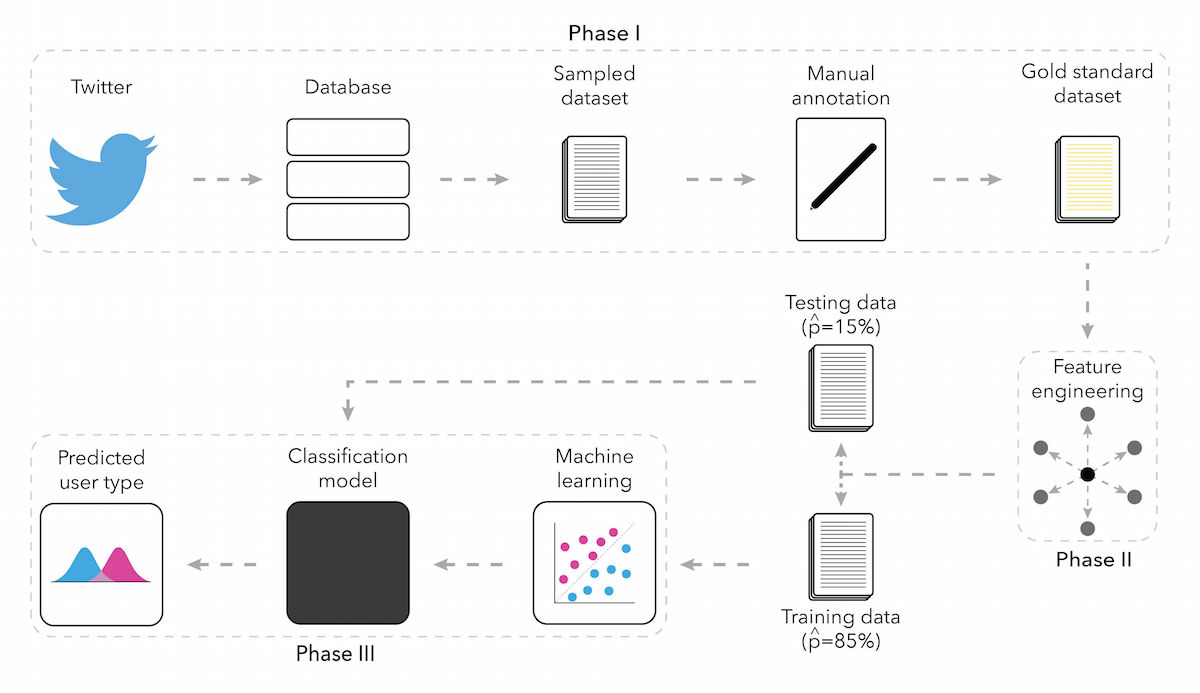
Approach to classifying Twitter users who tweet about e-cigarettes.

Using a supervised machine learning approach, we developed models to predict different types of Twitter users who tweet about e‑cigarettes. First, a random sample of Twitter handles that have tweeted about e‑cigarettes was obtained, and our trained human coders examined the handles’ profiles and manually labeled a specific user type for each handle. Next, Twitter metadata and a sample of tweets for each labeled user were gathered, and features that reflect users’ metadata and tweeting behavior were created. In the final steps, multiple machine learning algorithms to identify a model with the best classification performance were tested. Figure 1 illustrates our approach to developing the classification model, which we describe further in the sections below. This study was exempt from the institutional review board (IRB) review because it used publicly available Twitter data. Our approach to obtaining and analyzing the Twitter data was in compliance with Twitter’s terms of service at the time of the study, such as removing tweets that were deleted or made private by the user.

### Phase 1: Twitter Data Source and Manual Annotation of User Types

Using Twitter’s enterprise application programming interface (API) platform, Gnip, we collected e‑cigarette–related tweets posted between November 2014 and October 2016. A comprehensive search syntax was developed with 158 keywords, including terms such as *ecig*, *vape*, and *ejuice*, as well as popular e‑cigarette brands and hashtags, which resulted in approximately 11.5 million e‑cigarette–related tweets from 2.6 million unique users. Next, a random sample of the users associated with these tweets was reviewed, and the content of their posts was examined to identify the range of entities tweeting about e‑cigarettes. Using a grounded theory approach informed by literature review and guidance from subject matter experts, a protocol was developed for categorizing Twitter users who tweet about e-cigarettes according to the following types: (1) individual, (2) vaper enthusiast, (3) informed agency, (4) marketer, and (5) spammer (see Table 1).

Six coders were trained using the protocol and practice data to classify the user types manually. For each user, the coders reviewed the user’s profile page on Twitter, which included a profile description and a sample of recent tweets on their timeline, which may have included e‑cigarette and non-e‑cigarette topics. Random samples of Twitter users were double coded until at least 300 labeled cases were obtained per user type. Coding discrepancies were resolved by an adjudicator. In total, 4897 users were manually classified according to the user type definitions (see Table 1 for coding results).

**Table 1:** Manual classification of Twitter users who tweet about e-cigarettes: user type definitions and proportion of each type in manually labeled sample.

Type		Definition	Sample, N
Individual		The account of a real person whose Twitter profile information and tweets reflect their individual thoughts and interests. An individual is someone whose primary post content is not about vaping.	2168
Vaper enthusiast		The account of a person or organization whose primary content is related to promoting e‑cigarettes but is not primarily trying to sell e‑cigarettes or related products.	334
**Informed agency^a^**			622
	News media	The account of a newspaper, magazine, news channel, etc. News media does not include vaping-specific news sources.	
	Health community	The account of a public health organization, coalition, agency, or credible individual affiliated with an organization. These may also be the accounts of organizations with authority on a topic that should be thought of as *trusted sources*.	
**Marketer^a^**			752
	Marketer	An account marketing e‑cigarette or vaping products. These accounts can belong to a Web-based or brick-and-mortar retailer or an individual who is an affiliate marketer.	
	Information aggregator	An account that primarily aggregates information about e‑cigarettes/vaping and where most or all tweets are news articles related to e‑cigarettes/vaping. This account could also aggregate vaping coupons or deals.	
**Spammer**		An account that does not fall into one of the other coding categories. These accounts often post on a broad range of topics unrelated to this project, and their content can be nonsensical. Anecdotally, it was observed that many of these accounts exhibited *bot* behaviors.	1021

^a^During manual annotation of data, we initially categorized subtypes of informed agency (ie, news media and health community) and marketer (ie, marketer and information aggregator) user types, but we did not identify sufficient numbers of user handles for these subtypes to conduct meaningful analyses. Thus, during the feature selection and modeling phases, we collapsed across user subtypes to define five total user types.

### Phase 2: User Metadata Features and Derived Behavioral Features

Next, we built out the feature space for 4897 labeled users, extracting the metadata provided by the Twitter API and engineering our own features that were derived from the users’ tweet text (see Multimedia Appendix 1).

#### User Metadata Features

The Twitter API provides basic profile information about a user such as screen name, location, bio, number of friends, number of followers, and total number of tweets. The API also provides the actual tweet text and underlying metadata associated with tweet text that was used in this study to characterize the tweeting behavior (eg, retweet) of the users. These types of metadata features have a demonstrated utility in characterizing different types of users [30,31]. Using the Twitter API, 15 metadata features were obtained for each labeled user. Examples of metadata features include number of followers and the number of tweets favorited by the user (see Multimedia Appendix 1).

#### Derived Tweeting Behavior Features

In addition to the metadata, the users’ tweet text data were also examined to capture their tweeting behavior. It was hypothesized that tweeting behaviors would vary across different user types (eg, individuals are likely to tweet about more diverse topics than marketers). Studies have shown that linguistic content of social media posts is particularly useful because it illustrates the topics of interest to a user and provides information about their lexical usage that may be predictive of certain user types [32,33]. For each Twitter handle, the 200 most recent publicly available tweets were collected using the Twitter REST API. Previous studies have shown that 100 to 200 tweets are typically sufficient for predicting Twitter user characteristics [34,35]. These 200 tweets included tweets about e-cigarettes as well as non-e-cigarette–related topics. The non-e-cigarette–related tweets were included because most of the user types examined in this study (eg, news media agency, individuals, and public health agencies) do not tweet about e-cigarettes alone.

To capture the users’ tweeting behavior, 58 features derived from the behavioral and linguistic content of the account profile and the tweet text were generated; summary statistics of sets of users’ tweets were also created. To generate these features, a variety of text mining techniques were used to capture the distribution characteristics of the users’ tweeting behavior and word usage. For example, the minimum, maximum, median, mean, and mode for how many times an e‑cigarette keyword was used per tweet was calculated. A term frequency-inverse document frequency matrix of each user’s corpus of tweets (up to 200 tweets) was also created, and the pair-wise cosine similarity between each tweet was calculated. For each user, the mean and standard deviation of the set of cosine similarity values, which provided a sense of the semantic diversity and consistency of a user’s vocabulary, was calculated. After generating the behavioral features, we dropped nine features in our dataset that had more than 10% missing data. Then, a mean imputation was performed on the derived features that had 10% or less missing data.

### Phase 3: Predictive Models

To determine the best model for classifying the user types, several different algorithms were built and compared using the features described in phase 2. Before modeling, the data were split into a training set (85%) and a test set (15%), using stratified sampling to preserve the relative ratio of classes across sets. To construct our models, a stratified 10-fold cross-validation on the training set was first run and eight different classifiers as well as a *dummy classifier* were evaluated. The dummy classifier—which makes random guesses based on the known distributions of user types in the training data—served as a benchmark for evaluating the performance of our other models. The results from these analyses showed that *F*_1_ scores were highest (82.5%) for the Gradient Boosting Regression Trees (GBRT) classifier and lowest for the dummy classifier (28.6%) (see Multimedia Appendix 2 for results of all classifiers).

On the basis of these results, the GBRT algorithm was used to classify the testing dataset. The GBRT approach builds an additive model in a forward stage-wise fashion [36]. The *boosting* technique combines an ensemble of many weak predictive models—in this case, shallow trees—into a single strong one [37]. Each weak model is weighted and trained to be an *expert* on the residuals of the preceding model [38,39] (see Multimedia Appendix 3 for additional information about GBRT and the other algorithms examined).

To determine the best tuning values for the hyperparameters in our model, a fourfold grid-search cross-validation on the training dataset was run (see Multimedia Appendix 3). Then, to evaluate the performance of our tuned GBRT model and the marginal impact of our derived features in improving class differentiation, two separate models were run—one composed of metadata features alone and the other composed of both metadata and derived features. These two separate models were used to evaluate the marginal impact of adding derived features as metadata features for user profile and tweets are easily obtainable, whereas derived features are more labor intensive to create. Finally, the extent of misclassification and the most important features for user types were examined.

## Results

### User Classification Model Results

Table 2 presents the GBRT model results for predicting different types of Twitter users who have tweeted about e-cigarettes. When the complete dataset (metadata + derived features) was tested, the model achieved an average *F*_1_ score of 83.3% across all user types. The *F*_1_ score was highest for predicting individuals (91.1%) and progressively lower for informed agencies (84.4%), marketers (81.2%), spammers (79.5%), and vaper enthusiasts (47.1%).

The metadata-only model (72.7%) achieved lower *F*_1_ scores than the full model (83.3%) (Table 2). Including derived features in the full model improved classification results for each user type, with improvements in *F*_1_ scores ranging from 7.5% for individuals to 30.9% for vaper enthusiasts.

**Table 2:** Classification of Twitter users who tweet about e-cigarettes: Gradient Boosting Regression Trees (GBRT) results comparing full model and metadata-only model.

User type	Full model (metadata + derived data)			Metadata-only model		
	*F*_1_ score, %	Recall, %	Precision, %	*F*_1_ score, %	Recall, %	Precision, %
Individual	91.1	92.3	89.8	83.6	86.2	81.2
Vaper enthusiast	47.1	40.0	57.1	16.2	12.0	25.0
Informed agency	84.4	78.5	91.3	70.0	67.7	72.4
Marketer	81.2	85.9	77.0	65.6	72.6	59.9
Spammer	79.5	81.1	78.0	74.8	71.9	78.0
Average	83.3	83.7	83.3	72.7	73.7	72.3

### Misclassification

**Figure 2:**
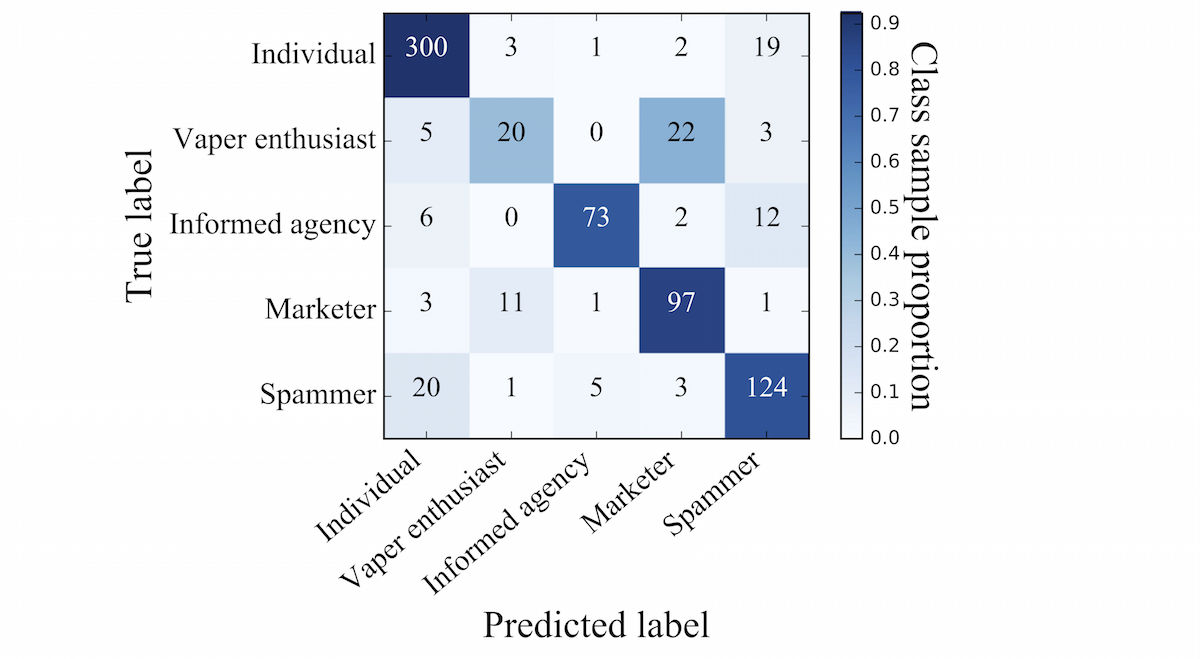
Distributions of manually labeled versus model-predicted classification of Twitter users who tweet about e-cigarettes.

To further examine variations in the predictive performance across user types, a confusion matrix illustrating predicted and actual user types was generated. Figure 2 shows the distribution of predicted user types on the horizontal axis and actual user types from the manual coding on the vertical axis. To aid in interpretation, the predicted sample proportion for each user type is shaded from light (low proportion) to dark (high proportion). Darker shading in the cells along the diagonal indicates correct classification, whereas darker shading elsewhere indicates misclassification. For example, of the 325 users manually coded as individuals, 300 (92.3%) were correctly predicted to be individuals. In contrast, there was a high level of misclassification of vaper enthusiasts; only 20 of the 50 vaper enthusiasts (40.0%) were correctly predicted to be vaper enthusiasts, whereas 22 (44.0%) were misclassified as marketers.

**Figure 3:**
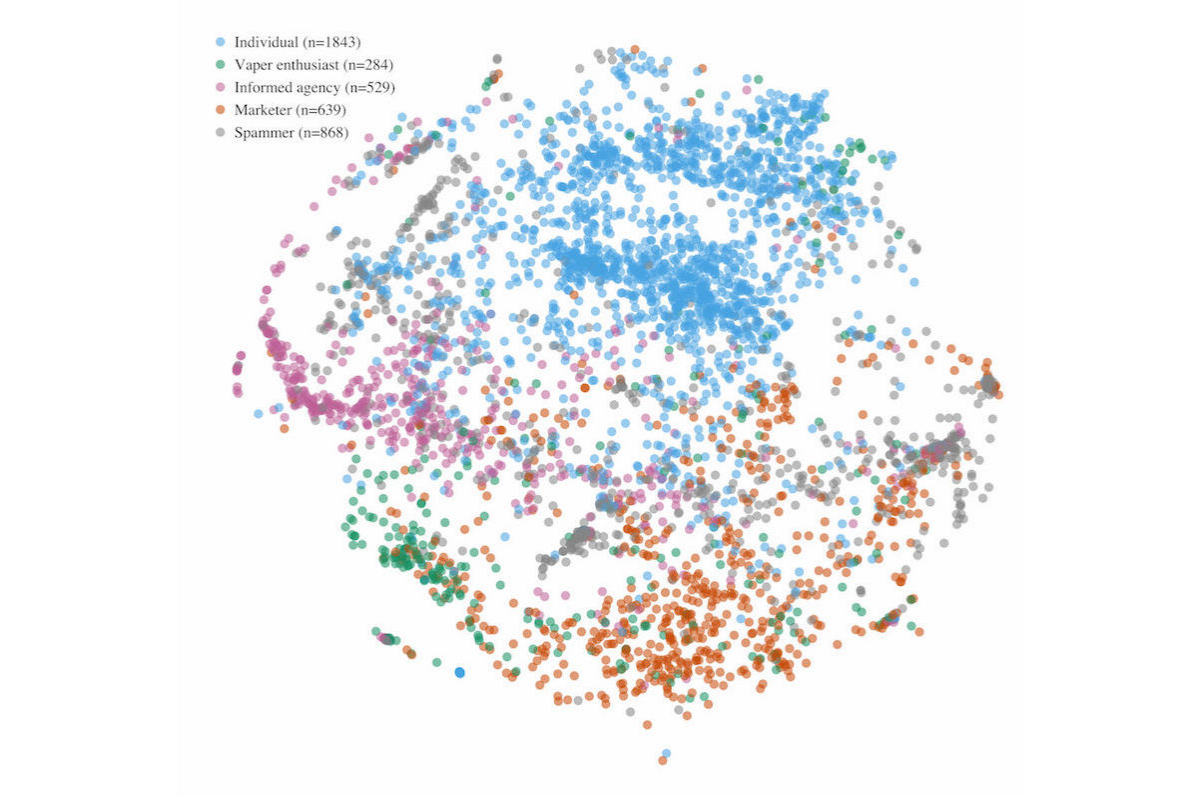
Two-dimensional t-SNE visualization of Twitter users who tweet about e-cigarettes.

A two-dimensional (2D) plot of the feature space was also constructed to better understand the extent to which the user types fall into naturally separated clusters (see Figure 3). T­­o accomplish this, a dimensionality reduction method called t-distributed stochastic neighbor embedding (t-SNE) [40] was used to create a 2D representation of the 78-dimensional feature space (see Figure 3). The results of the t-SNE plot indicate that individuals, marketers, and informed agencies fall into fairly discrete clusters, with some users in each class falling closer to other clusters. The plot also shows that whereas spammers are also fairly distinct from other user types, this user type appears to comprise two to three clusters, perhaps suggestive of different subtypes of spammers. Vaper enthusiasts also comprise a distinct cluster, but there appears to be a substantial overlap between vaper enthusiast and marketer clusters.

**Table 3:** Ten most important features in predicting Twitter users who tweet about e-cigarettes across all user types.

Features^a^	Proportion of feature importance among all variables, %
Statuses count	5.1
Followers count	4.1
Original tweet raw keyword count	3.7
Profile description keyword count	3.3
Original tweet cosine similarity mean	3.2
Retweet cosine similarity mean	3.0
Friends count	3.0
Retweet raw keyword count	3.0
Listed count	2.9
Original tweet URL count mean	2.7
Favorites count	2.7

^a^Most important feature among each user type—Individual: favorites count (4.9%); Vaper enthusiast: retweet raw keyword count (8.3%); Informed agency: followers count (6.5%); Marketer: original tweet raw keyword counts (8.9%); Spammer: statuses count (8.1%).

**Figure 4:**
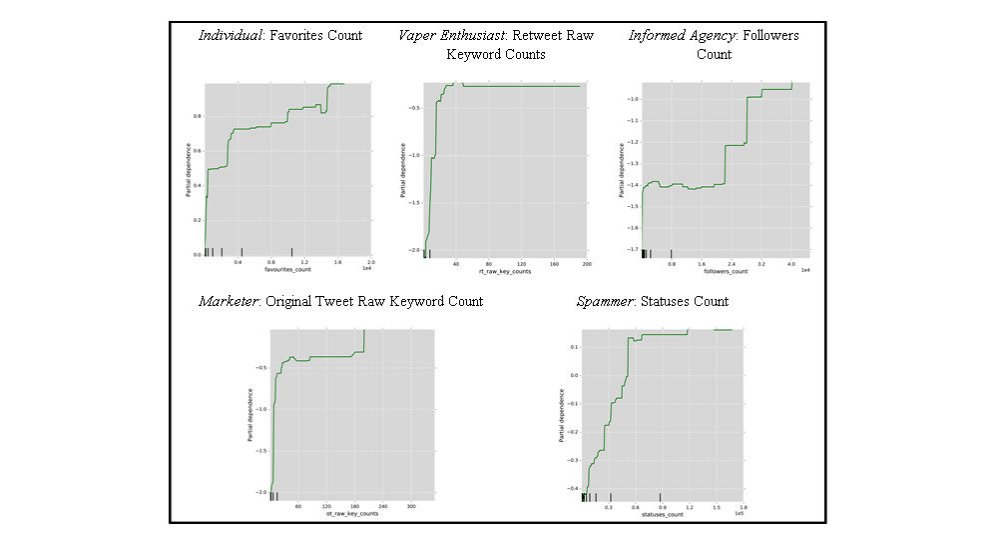
Partial dependence plots of top features by user type for users who tweet about e-cigarettes.

### Feature Importance

To better understand the contribution of each variable in our modeling outcome, each variable was evaluated using Gini Importance, which is commonly used in ensembles of decision trees as a measure of a variable’s impact in predicting a label that also takes into account estimated error in randomly labeling an observation according to the known label distributions [41]. Table 3 shows the top 10 most important features, ranked by the proportion of feature importance among all variables in the full model. Results show that two profile metadata features—statuses count and followers count—represent the most important features in the model, with values of 5.1% and 4.1%, respectively. Several derived data features were also important, including original tweet raw keyword counts (3.7%), profile description keyword count (3.3%), and original tweet cosine similarity mean (3.2%). The single most important feature varied among the user types. For individuals, the most important feature was favorites count (4.9%); for vaper enthusiasts, it was retweet raw keyword count (8.3%); for informed agencies, it was followers count (6.5%); for marketers, it was original tweet raw keyword count (8.9%); and for spammers, it was statuses count (8.1%). Feature importance scores for all features examined is available in Multimedia Appendix 4.

Partial dependence plots (PDPs) illustrate the dependence between a target function (ie, user type) and a set of target features. Figure 4 shows PDPs for each user type, illustrating the association between user type and the most important feature for that particular group. Figure 4 shows the most important features for each user type, whereas Table 3 summarizes the most important features across all user types. For individuals, as the number of tweets the user has liked increases, a given user is more likely to be classified as an individual. For informed agencies, as the number of followers increases, a given user is more likely to be classified as an informed agency. For marketers, as the number of raw keyword counts increases in a given user’s set of original tweets, that user is more likely to be classified as a marketer. This indicates that marketers tend to create original content using e‑cigarette terms. For spammers, as the total number of statuses (original tweets and retweets) count increases, a given user is more likely to be classified as a spammer. For vaper enthusiasts, as the number of raw keyword counts increases in a given user’s set of retweets, that user is more likely to be classified as a vaper enthusiast. This indicates that vaper enthusiasts tend to retweet content with e‑cigarette terms.

## Discussion

### Principal Findings

In summary, we developed algorithms with relatively high performance in predicting different types of Twitter users that tweet about e‑cigarettes. The rates of precision and recall for *most* user types ranged from 78% to 92%, which was well above the baseline dummy classification and serves as a new baseline for the future user type classification of users who tweet about e‑cigarette content on Twitter. Although using metadata features alone in user classification demonstrates performance gains over dummy classification, the results of this study suggest that including additional tweet-derived features that capture tweeting behavior significantly improves the model performance—an overall *F*_1_ score gain of 10.6%—beyond metadata features alone. Previous studies have shown that tweet linguistic patterns are strong predictors of social media user demographics [42]. This is the first study to show the predictive utility of tweeting behavior in classifying different types of users who tweet about e‑cigarettes.

We achieved the best performance in predicting individuals, informed agencies (news media and health agencies), and marketers. In contrast, vaper enthusiasts were challenging to predict and were commonly misclassified as marketers. There are several reasons why this may be the case. First, it is possible that there were not enough labeled cases of vaper enthusiasts for the machine learning models; there were only 334 labeled cases of vaper enthusiasts (6.8% of all labeled users) compared with 622 to 2168 cases for the other classes. Second, vaper enthusiasts are an evolving group of individuals, and their tweeting behavior may therefore vary more than other established user types such as informed agencies (eg, news media and health agencies). Third, our definition of vaper enthusiasts may not have been distinct enough from marketers; a vaper enthusiast was defined as a user whose primary objective is to *promote but not sell* e‑cigarette/vaping products, whereas a marketer was defined as a user whose primary objective is to *market and sell* e‑cigarette/vaping products. The distinction of promoting but not selling may have been too subtle to pick up, as vaper enthusiasts promote e‑cigarettes by using similar strategies that marketers employ to sell products, such as sharing information about new products, promoting giveaways, and posting product reviews. It is possible that having more labeled cases and extracting more than 200 tweets per handle could improve model performance and better discriminate vaper enthusiasts from marketers. Alternatively, not being able to distinguish vaper enthusiasts from marketers may signal that they share common interests and possible affiliations. With the rise of *social influencer marketing*, where brands incentivize influencers to promote products or subcultures on social media, it is possible that vaper enthusiast messaging may represent commercial marketing interests. The vagueness and ambiguity that was observed between the feature spaces of the vaper enthusiast and marketer classes warrants additional research that examines potential relationships between vaper enthusiasts and e-cigarette commercial entities.

Given the overlap between vaper enthusiasts and marketers, a possible strategy to improve predictive performance might be to combine the two groups. In fact, in their study, Kavuluru and Sabbir [27] classified e‑cigarette proponents as “tweeters who represent e‑cigarette sales or marketing agencies, individuals who advocate e‑cigarettes, or tweeters who specifically identify themselves as vapers in their profile bio.” They achieved a high level of accuracy in predicting these e‑cigarette proponents (97% precision, 86% recall, and 91% F-score). Although combining these groups may help improve model performance, from a public health perspective, these are distinct groups whose Web-based behaviors have different implications for regulatory agencies. For example, FDA has the authority to regulate claims made by e‑cigarette companies and will need to monitor e‑cigarette brand social media handles to ensure that they are being compliant with regulatory policies (eg, not making cessation claims, posting warning statements about the harmful effects of nicotine) [43]. In contrast, FDA cannot regulate claims made by vaper enthusiasts because they are individuals and not companies selling e‑cigarette products. Therefore, distinguishing vaper enthusiasts from marketers is critical to informing FDA compliance and enforcement efforts. Being able to distinguish vaper enthusiasts from marketers is also important with regard to public health education efforts because vaper enthusiasts have been known to undermine e‑cigarette education campaigns. For example, when the California Department of Public Health launched its *Still Blowing Smoke* campaign to educate consumers about the potential harmful effects of e‑cigarette use, vaper advocates launched a countercampaign (*Not Blowing Smoke*). By using both hashtags and creating new accounts, the countercampaign attacked the credibility of messages of the California Department of Public Health and effectively controlled the messaging on social media [44]. We would argue that classifying marketers and vaper enthusiasts separately is important for informing e‑cigarette surveillance, regulatory, and education efforts; thus, future studies should build on our results and examine methods to improve classification of vaper enthusiasts.

In this study, the top features that were most predictive of each user type were also examined. Individuals like more tweets than nonindividuals; informed agencies have more followers than their counterparts; marketers use more e‑cigarette words in their original tweets than nonmarketers; vaper enthusiasts retweet e‑cigarette content more than nonvaper enthusiasts; and more frequent tweeting behavior is indicative of spammers. Given the infancy of this research, the findings of this study should be viewed as an initial inquiry into classifying different types of users who tweet about e‑cigarettes. Future studies should build on this work and examine other features that may be predictive of these classes of users. For example, other researchers have examined features such as sentiment of tweets [27] to classify certain subgroups of users who tweet about e‑cigarettes.

### Limitations

Our study has several limitations. First, because of resource constraints, we only collected the 200 most recent tweets for the users in our dataset, and some users had less than 200 tweets in total. Previous studies examining Twitter metadata and linguistic features to predict sociodemographic characteristics of users (eg, gender and age) have extracted up to 3200 tweets per handle, but other researchers have also found that having more than 100 tweets per handle did not necessarily improve the model performance [34]. Additional studies are needed to determine whether increasing the number of tweets for each user would increase the importance of the behavioral features in our classification of user types. Second, the methodology involved manual feature engineering, which can be time intensive and is limited to researcher-defined categories. A neural network approach could enable more automated construction of other text-based features that may help in distinguishing user types. Whereas computational text mining methods make it easy to create a multitude of different features, having more features may not necessarily yield information that is useful for classification tasks [31]. Furthermore, issues about scalability and reproducibility should be considered. As social media data are increasingly being used in applied fields such as public health, we need to consider how to balance the resources to conduct this type of analysis with a high level of accuracy and methodological rigor against timeliness and usefulness of the data to inform surveillance and regulatory efforts. Third, the definitions used to classify Twitter users who tweet about e‑cigarettes may not be generalizable. Some of the methodologies would be applicable in other contexts (eg, identifying marketers in other domains), but results may not generalize readily across domains.

### Comparison With Prior Work

This is the first study we are aware of that has examined methods to predict a broad set of different types of users tweeting about e‑cigarettes. Previous studies have examined either the topic of e‑cigarette tweets [23,24] or a single user type (eg, proponents of e‑cigarettes vs nonproponents) [27]. In this study, five different categories of users who were involved in public discourse about e‑cigarettes and groups that are of interest to inform public health surveillance, education, and regulatory efforts were examined. Second, multiple machine learning algorithms were tested and GBRT was used, which has not been used previously for this purpose. This is important, given the limited work in this area and the lack of existing methodology to build on. Third, in addition to analyzing Twitter metadata features, as prior studies have done, behavioral features that are shown to be important in performance gains were also examined. Finally, by using PDPs, evidence for how important features relate to a given user type was also provided.

### Conclusions

In conclusion, this study provides a method for classifying five different types of users who tweet about e‑cigarettes. Our model achieved high levels of classification performance for most groups; examining tweeting behavior was critical in improving the model performance. The results of our approach can help identify groups engaged in conversations about e‑cigarettes online to help inform public health surveillance, education, and regulatory efforts. Future studies should examine approaches to improve the classification of certain user groups that were more challenging to predict (eg, vaper enthusiasts).

## Appendix

Multimedia Appendix 1 List of Twitter metadata features and derived behavioral features used in models to classify Twitter users who tweet about e-cigarettes.

Multimedia Appendix 2 Modeling results of different machine learning algorithms to classify Twitter users who tweet about e-cigarettes.

Multimedia Appendix 3 Overview of machine learning algorithms examined.

Multimedia Appendix 4 Importance of features in models to predict Twitter users who tweet about e-cigarettes.

## Abbreviations

API: application programming interface
FDA: Food and Drug Administration
GBRT: Gradient Boosted Regression Trees
IRB: institutional review board
PDPs: partial dependence plots
t-SNE: t-Distributed Stochastic Neighbor Embedding
2D: two-dimensional
