# Supplementary material for: Classification of Twitter Users Who Tweet About E-Cigarettes
Source: JMIR Public Health Surveill. 2017 Sep 26;3(3):e63. doi: 10.2196/publichealth.8060 (PMC5635233; doi:10.2196/publichealth.8060)
Supplement: Multimedia Appendix 1 [file publichealth_v3i3e63_app1.pdf]

Supplementary Table 1. List of Twitter metadata features and derived behavioral features used in models to classify Twitter users who tweet about e-cigarettes.

Metadata features (N=15)
Contributors enabled
Default profile
Default profile image
Favorite count (How many times the tweet has been liked by Twitter users)
Favorites count (Number of tweets this user has liked over the account's lifetime)
Followers count
Friends count
Geo enabled
Is translator (if user is a participant in Twitter's translator community)
Listed count (Number of lists the account is added to by other twitter users)
Profile background tile
Profile use background image
Retweet count
Statuses count
Verified (Whether or not the account is verified)

Derived behavioral features[a] (N=58)
Original tweet count
Original tweet favorite count
Total hashtag count in original tweets
Total URL count in original tweets
Total mention count in original tweets
Mean hashtag count in original tweets
Mean URL count in original tweets
Mean mention count in original tweets
Median hashtag count in original tweets
Median URL count in original tweets
Median mention count in original tweets
Max hashtag count in an original tweet
Max URL count in an original tweet
Max mention count in an original tweet
Minimum hashtag count in an original tweet
Minimum URL count in an original tweet
Minimum mention count in an original tweet
Original tweet mean cosine similarity
Original tweet cosine similarity standard deviation
Total keyword count in original tweets
Unique keyword count in original tweets
Total keyword count in hashtags in original tweets
Unique keyword count in hashtags in original tweets

Total keyword count in URLs in original tweets
Unique keyword count in URLs in original tweets
Total keyword count in mentions in original tweets
Unique keyword count in mentions in original tweets
Language count in original tweets
Screen name keyword count
Description keyword count

[a] We also examined retweets of all tweet-related behavioral features (i.e. first 28 features listed in table). 28 features * 2 (tweets and retweets) = 56 + last 2 features in table = 58 total behavioral features.
