# Supplementary material for: Classification of Twitter Users Who Tweet About E-Cigarettes
Source: JMIR Public Health Surveill. 2017 Sep 26;3(3):e63. doi: 10.2196/publichealth.8060 (PMC5635233; doi:10.2196/publichealth.8060)
Supplement: Multimedia Appendix 2 [file publichealth_v3i3e63_app2.pdf]

Supplementary Table 2. Modeling results of different machine learning algorithms to classify Twitter users who tweet about e-cigarettes.

Model	F_1 score (standardized)
Gradient Boosting Regression Trees	82.5%
Support Vector Machine	80.5%
Logistic Regression	79.9%
Random Forest	79.5%
K-Nearest Neighbors	76.9%
AdaBoost	76.7%
Naïve-Bayes	72.7%
Decision Trees	71.6%
Dummy Classifier	28.6%
