# Supplementary material for: Classification of Twitter Users Who Tweet About E-Cigarettes
Source: JMIR Public Health Surveill. 2017 Sep 26;3(3):e63. doi: 10.2196/publichealth.8060 (PMC5635233; doi:10.2196/publichealth.8060)
Supplement: Multimedia Appendix 3 [file publichealth_v3i3e63_app3.pdf]

Supplementary Appendix. Overview of machine learning algorithms examined.

Overall, we decided to go with the gradient boosting regression trees (GBRT) algorithm in our analysis because it had the best results, we were interested in exploring nonlinear relationships in partial dependence plots, and a trained GBRT model runs quickly, which would be useful if we wanted to have a near real-time classifier running on new tweets that we may pull in. Moreover, GBRT can be very powerful and flexible, and we have seen little use of it in the literature of this domain.

The following is a high-level overview of the models we examined. Many of the descriptions were adapted from scikit-learn.org, which is also the source of the algorithm implementations that we used. More in-depth details can be found there.

- **GBRT** and **AdaBoost** are examples of ensemble "boosting" methods where many "weak" (i.e., slightly better than random guessing) trees are combined to produce a single "strong" model. GBRT, in particular, handles mixed-data well, and it tends to be robust to outliers. One drawback is that training a GBRT model can be time-intensive, but once it is trained, it runs quickly.
- A **random forest** is an example of an ensemble "averaging" method that fits many decision trees to subsamples of the data. It averages the results of these trees, making it resilient to over-fitting.
- **Support vector machines (SVMs)** are a modeling approach that attempts to differentiate classes of observations by finding "hyperplanes" between points in n-dimensional space. For a given observation, that observation's value for any given variable can be thought of as a coordinate in space. Thus, data occupy this full feature space, and SVMs try to find a boundary between observations of different classes. For example, if one imagines a box as the entire feature space, one could divide the box with a flat piece of paper, where the paper is a hyperplane that has fewer dimensions than the box.
- **Naïve-Bayes** is a classifier that attempts to apply Bayes' theorem "naively" in the sense that it assumes independence between variables. While the naïve assumption may be blatantly false, this classifier has a history of working well and has been used for things like document classification and spam detection. Moreover, they can be trained with smaller datasets, and they can handle higher dimensional spaces well.
- **K-Nearest Neighbors (k-NN)** is a simple algorithm that attempts to classify observations based on the classes of that observation's neighbors. The k-NN algorithm can be sensitive to the local structure of the data (i.e., the structure of data close to each other in feature space), depending on how close and distant neighbors are weighted.
- A **decision tree** is a nonparametric modeling approach that attempts to predict the value of a target response variable by learning rules about associations between independent variables in the data and the target variable. Decision trees are able to handle mixed data (i.e., numerical and categorical data).

- **Logistic regression** is a modeling approach that attempts to use a linear function to fit a line in the data (often using least squares) that separates classes in the data. Unlike linear regression, the response variable in logistic regression is categorical.
- A **dummy classifier** is a modeling approach that makes class assignments to data. This approach can be implemented in various ways. In our case, we made random guesses based on known distributions of classes in the data. This gives us a benchmark on how one would perform on classification if they simply guessed.
