# Supplementary figures and images for: Classification of Twitter Users Who Tweet About E-Cigarettes
Source: JMIR Public Health Surveill. 2017 Sep 26;3(3):e63. doi: 10.2196/publichealth.8060 (PMC5635233; doi:10.2196/publichealth.8060)

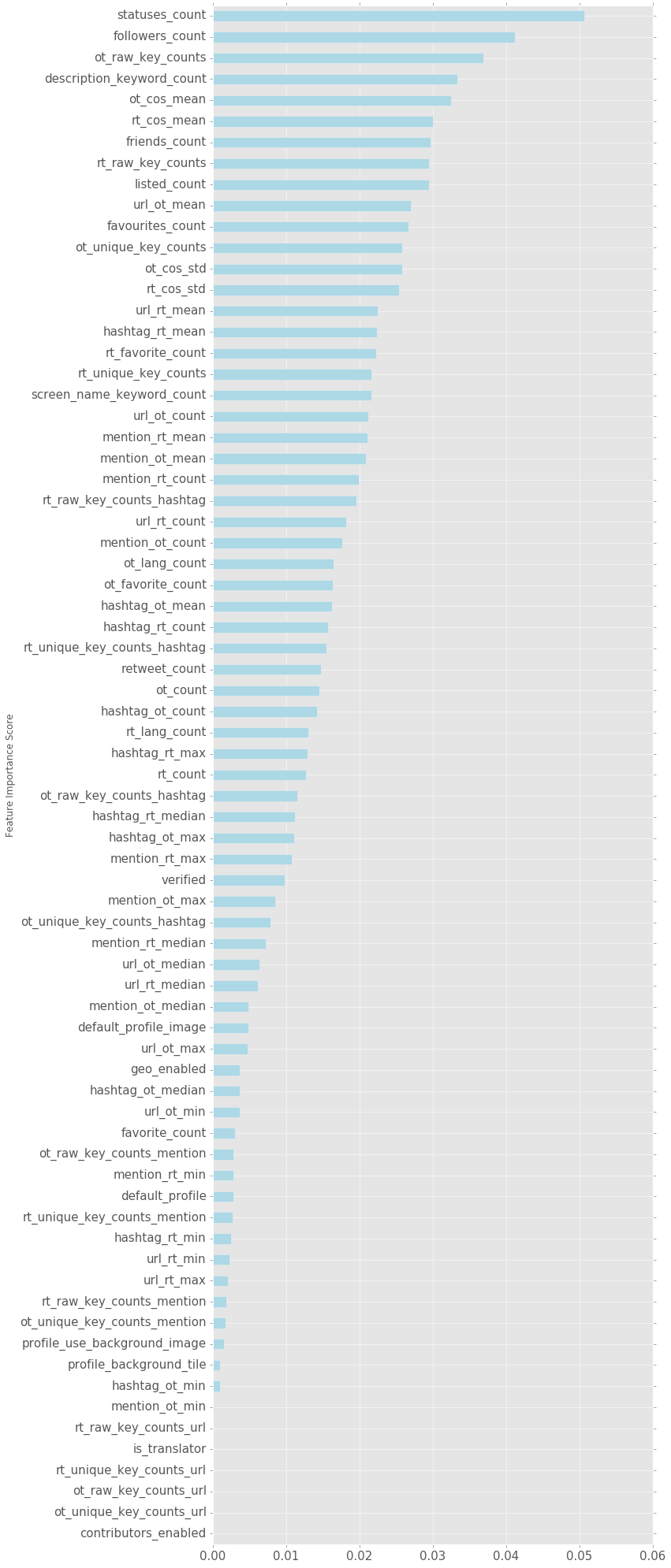

Supplement: Multimedia Appendix 4 [file publichealth_v3i3e63_app4.png]
